# Effectiveness and safety of warm needle acupuncture on chronic renal failure: Protocol for a systematic review and meta-analysis

**DOI:** 10.1097/MD.0000000000018706

**Published:** 2020-01-10

**Authors:** Cihui Huang, Yunxin Lin, Yaqin Yang, Fangdong Zeng, Huaqing Jiang, Ting Lin, Liang Zheng

**Affiliations:** a The First Clinical Medical; b The Fourth Clinical Medical School of Guangzhou; c Acupuncture and Manipulation Center, The First Affiliated Hospital of Guangzhou University of Chinese Medicine, Guangzhou, China.

**Keywords:** chronic renal failure, protocol, systematic review, warm needle acupuncture

## Abstract

**Background:**

Warm needle acupuncture (WNA) is an integral part of the acupuncture therapy. Chronic renal failure (CRF) is a common disease, which is a type of kidney disease characterized by a slow and progressive decline in renal function. The clinical practice indicates that WNA has a therapeutic effect on CRF. Therefore, we will provide a protocol to explore the effectiveness and safety of WNA for CRF.

**Methods:**

We will search the randomized controlled trials literatures of WNA for CRF in 5 English databases (PubMed, Web of Science, EMBASE, the Cochrane Central Register of Controlled Trials [Cochrane Library], and World Health Organization International Clinical Trials Registry Platform) and 4 Chinese databases (Chinese national knowledge infrastructure, Chinese Scientific Journal Database Information, Wanfang Database, and Chinese biomedical literature database). The renal function will be considered as the primary outcome and the secondary outcome will include curative effect, security, syndrome according to standards for assessing traditional Chinese medicine, adverse events caused by WNA, such as dizziness, nausea, vomiting, weariness, and so on. We will use EndnoteX7 software to perform the selection of the studies. And all analyses will be conducted by using RevMan software V5.3.

**Result:**

This study will provide a rational synthesis of current evidences for WNM on CRF.

**Conclusion:**

The conclusion of this study will provide evidence to judge the effectiveness and safety of WNA on CRF.

**Registration:** PROS-PERO CRD42019144530

## 1 Introduction

Chronic kidney disease (CKD) has the characteristics of high prevalence, poor prognosis, and high medical expenses. It has become recognized as an important disease that endangers human health across the world. Chronic renal failure (CRF) is a clinical syndrome in the later stage of CKD. CRF is a type of kidney disease characterized by a slow and progressive decline in renal function.^[1]^ CRF has many complications, which seriously affect the quality of life and longevity of patients. Nowadays the morbidity and mortality of CRF is rising markedly, which has brought a heavy financial burden in the world.^[2,3]^ In 2012, there are 119.5 million CRF patients in China.^[4]^ Therefore, how to delay the progression of CRF, reduce the harm of complications and improve the quality of life of patients has important academic and social significance.^[5]^ It is imperative to develop new and effective agents from herbs or edible medicinal materials to treat or alleviate CRF. The main therapeutic modalities of CRF are medicine, hemodialysis, and kidney transplantation, but these have some side effect like tolerance and dependence.^[6]^ In general, neither of the methods is perfect and both have their own defects. Therefore, numerous research studies have been carried out on this topic to reduce the symptoms of CRF in patients with efficiency and safety.

As one of the most characteristic therapy of traditional Chinese medicine (TMC), acupuncture is common in many countries, not just in China. Acupuncture has the advantages of simple operations and significant curative effect, which is used to treat diseases by inserting the needle into specific points in the body. Many researches have indicated that acupuncture therapy serve the special and obvious function of treating many diseases.^[7–8]^ Warm needle acupuncture (WNA) is an important component of the acupuncture therapy. Acupuncturists add warm stimulation, such as the burning moxa to the handle of the needles, then heat is transmitted into deep tissues, so as to increase the therapeutic effect. It has combining the advantages of both acupuncture and moxibustion, WNA can produce better effectiveness. What's more, it has virtually no side effects.^[9]^ At present, some literatures has proved that WNA contributes to the treatment and recovery of disease.^[10–12]^

For instance, WNA therapy is applied in treating chronic pain caused by related diseases, including rheumatoid arthritis, joint pain, and cold or numb limbs. Electrical heat stimulation of the acupoints is effective in the treatment of low back pain.^[13]^

However, there have not been related reports on the effect of WNA on CRF. Therefore, our purpose of this review was to carry a systematic review and meta-analysis to evaluate the effectiveness and safety of WNA on CRF.

## 2 Methods

### 2.1 Protocol and registration

The protocol and search strategy of the review was registered in the International Prospective Register of Systematic Reviews database on October 28th 2019 (registration No. CRD42019144530). It was conducted and reported according to the preferred reporting items for systematic reviews and meta-analyses protocols (PRISMA-P) guideline.

### 2.2 Eligibility criteria

#### 2.2.1 Types of studies

All available randomized controlled trials (RCTs) on WNA for CRF will be included. Others such as case report, retrospective study, review, and studies which uses inappropriate random sequence generation methods will be excluded. There is no unified requirement on the blinding and language of the findings.

#### 2.2.2 Types of participants

##### 2.2.2.1 Diagnostic criteria^[14]^

(1) Endogenous creatinine clearance rate (CCR) < 80 mL/min.
(2) serum creatinine (Scr) > 133 umol/L.
(3) Has a history of chronic kidney disease or systemic disease involving the kidney.

##### 2.2.2.2 Inclusion criteria

(1) Patients accords with the diagnosis of the above diseases;
(2) Choose CRF as early and middle stage patients (Scr 133–442umol/L) creatinine clearance rate (Ccr) 20 to 80 mL/min;
(3) Nondialysis patients with stable condition were included in the observation of reversible factors such as pre-heart failure, infection, and hypovolemia;
(4) Age 20 to 60 years.

##### 2.2.2.3 Exclusion criteria

(1) Pregnant or lactating women;
(2) Complicated with serious primary diseases such as heart, brain, liver, and hematopoietic system;
(3) Psychiatric patients, who can not cooperate with the treatment;
(4) Age more than 60 years or less than 20 years.

#### 2.2.3 Types of interventions

The purpose of the study is on clinical trials of WNA for CRF. We will treat intervention group with WNA regardless of the form, type of needle, length of needle. The intervention group can combine other conventional treatments; while control group will only include only other conventional treatments.

#### 2.2.4 Outcome measures

##### 2.2.4.1 Primary outcome

The primary outcome measure is renal function: blood urea nitrogen, uric acid, Ccr, Scr.

##### 2.2.4.2 Secondary outcomes

(1) Curative effect,
(2) Security,
(3) Syndrome according to standards for assessing TCM,
(4) Adverse events caused by WNA, such as dizziness, nausea, vomiting, weariness, and so on.

#### 2.2.5 Exclusion criteria

In the study, if the following situations occur repeated test and no test data required by this program are available, acquired study will be excluded.

### 2.3 Data sources

#### 2.3.1 Electronic search

We will search relevant RCTs from inception to July 20th, 2019 in these databases such as Cochrane library, Embase, PubMed, Chinese Scientific Journal Database, Chinese National Knowledge Infrastructure, Chinese Biomedical and Literature Database, and Wanfang database. We search the retrieval type are “warm needle acupuncture” or “needle warming moxibustion” and “chronic renal failure.” In addition, endnote software 8.1 will be used to exclude the duplicate articles.

#### 2.3.2 Additional search

We will retrieve other potential articles in the reference list of retrieved studies to ensure comprehensiveness of this research. For some articles that are not included in the electronic database or related papers and journal, further consultation will be needed.

### 2.4 Study selection and Data extraction

**Figure 1:**
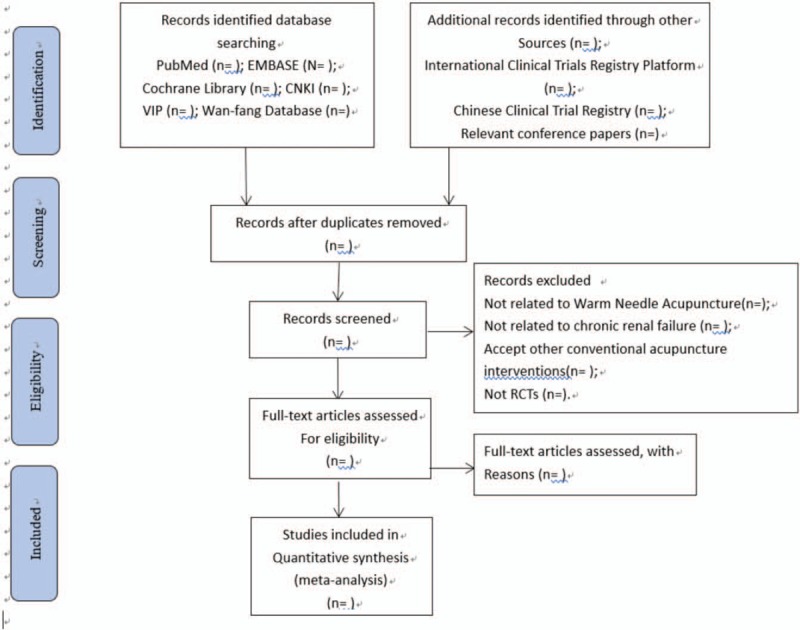
Flow diagram of study selection process. CNKI = Chinese National Knowledge Infrastructure; RCTs = randomized controlled trials, VIP = Chinese Science and Technology Pericodicals Database.

Two reviewers will first acquire useful findings with Endnote software 8.1 by reading abstracts of articles obtained from databases mentioned above. Then we will decide the final eligibility as a second analysis by reading through the whole findings. Disagreements will be solved by discussing with other researchers. In order to make clear the study selection procedure, PRISMA^[9]^ flow chart is shown (Fig. 1).

And then the following data will be extracted:

(1) Basic information (eg, author, publication year, etc);
(2) Interventions in control group and treatment group;
(3) Essential information (eg, number of participants, gender, mean and standard deviation for age, etc);
(4) Outcome measures;
(5) Random method.

### 2.5 Data analysis

#### 2.5.1 Risk of bias in the included studies

We will assess the risk of bias of each included RCT by using the Cochrane Handbook, version 5.1, which includes random allocation concealment, sequence generation, blinding of outcome assessments, incomplete outcome data, and selective outcome reporting, and so on. The result is low, high, or unclear after the evaluation of the risk of bias in each RCT. Two reviewers will independently perform evaluations of the methodological quality of each included study, and discuss when meets discrepancies. We will estimate the quality of the inclusions according to the modified Jadad rating scale.

#### 2.5.2 Statistical analysis

We will handle respectively meta-analysis and test sequential analysis of the included studies with statistical software (RevMan software V5.3). We choose mean difference and 95% confidence intervals (CIs), as to evaluating the continuous variables. For dichotomous variables, we adapt rate ratio and 95% CIs that used to evaluate the extracted data.

#### 2.5.3 Data synthesis

We can analyze useful data which based on the results of heterogeneity test with RevMan software V5.3, if there exists small or medium heterogeneity (*I*^2^ < 50%), we will choose fixed effect model analysis; but a random effect model analysis will be chose, if heterogeneity is significant (*I*^2^ > 50%).

#### 2.5.4 Assessment of heterogeneity

Heterogeneity was assessed by *I*^2^ test statistics. The random-effects model was used to analyze data if there was significant heterogeneity (*I*^2^ > 50%) between studies; otherwise, the fixed-effects model was used.

#### 2.5.5 Subgroup analysis

As for age, interventions, controls, and population area, we will conduct subgroup analyses. It means significant, if heterogeneity is ≥50%.

#### 2.5.6 Assessment of publication bias

The possibility of publication bias was assessed using a funnel plot of relative risk against the standard error for each trial. Asymmetries were assessed further with the Egger tests, with significant publication bias defined as a *P* value < .05, and visual inspection of an Egger funnel plot.

#### 2.5.7 Sensitivity analysis

It was used to test the stability of the results. If the heterogeneity is high, we will perform a sensitivity analysis based on the type of study, sample size, and methodological quality.

#### 2.5.8 Ethics and dissemination

Ethical approval will not be necessary because the data included in our study are derived from published literature and are not linked to individual patient data. The systematic review providing implication of the effectiveness and safety of WNM for CRF will be published in a peer-reviewed journal or conference presentations.

## 3 Discussion

CRF is a progressive injury, which is the final outcome of the development caused by various kidney diseases simultaneously. Patients with CRF who integrate with multiple symptoms such as gastrointestinal reactions, severe anemia, and renal hypertension are extremely common, which lead to cardiovascular system involvement as well as heart failure.^[15]^ In spite of replacement therapies and organ transplantation are made progress remarkably for the treatment of CFR, a growing number of issues take place in addition. For instance, an increased risk of adverse events negative effects could not be ignored, let alone the impact on the inferior quality of life being of significance to patients. Consequently, another preferential therapy with higher efficiency and less adverse events should be taken into consideration.

WNA, which also known as warm needle, is a combination of acupuncture and moxibustion. Nevertheless, it not only plays an essential role of TCM, but also takes advantages of both therapies. We can draw a definitive conclusion that WNA can enhance immunity, improve the circulation of blood, prevent diseases, adjust the overall condition of the human body and is beneficial to analgesic effect.^[16]^ Moreover, WNA is extremely safe that it operates on heating and stimulating acupoints which bring few side effects.

From what has been reported, this protocol might be a promising review of WNA on CRF for the first time. In virtue of evidence on the effectiveness and safety of WNA is insufficient and the potential mechanisms are indistinct. Thus, we intend to perform a systematic review and meta-analysis derived from the included literature, in order to provide referable evidence for clinical practice of the WNA on CRF, and make it come into public sight more extensively.

Despite the advantages of this study, several limitations should be acknowledged. To begin with, the sample size used by some trials seems small. Next in importance, some significant methodological deficiencies also exists.

In brief, in spite of the inadequacies, WNA should have beneficial effects on patients with CRF. Further rigorous studies are necessary to be developed for clinical research.

## Author contributions

**Conceptualization:** Cihui Huang, Yaqin Yang.

**Data curation:** Fangdong Zeng.

**Formal analysis:** Huaqing Jiang.

**Methodology:** Cihui Huang, Liang Zheng.

**Resources:** Liang Zheng.

**Software:** Ting Lin.

**Supervision:** Liang Zheng.

**Writing – original draft:** Cihui Huang, Yunxin Lin.

**Writing – review and editing:** Cihui Huang, Yunxin Lin, Yaqin Yang.

## References

[1] Zaki MR Ghazanfar A Hussain S Presentations, etiology and outcome of patients with chronic renal failure admitted at urology department, Mayo Hospital Lahore: a retrospective analysis of 1257 patients over a period of 10 years. Ann King Edw Med Univ 2003;9:58–61.

[2] Khoury T Tzukert K Abel R The gut-kidney axis in chronic renal failure: a new potential target for therapy. Hemodial Int 2017;21:323–34. 27634464 10.1111/hdi.12486

[3] Gilbertson DT Liu J Xue JL Projecting the number of patients with end-stage renal disease in the United States to the year 2015. J Am Soc Nephrol 2005;16:3736–41. 16267160 10.1681/ASN.2005010112

[4] Liu ZH Nephrology in China. Nat Rev Nephrol 2013;9:523–8. 23877587 10.1038/nrneph.2013.146

[5] Weldegiorgis M Smith M Herrington WG Socioeconomic disadvantage and the risk of advanced chronic kidney disease: results from a cohort study with 1.4 million participants. Nephrol Dial Transplant 2019;23:10.1093/ndt/gfz059 31329936

[6] Eirin A Lerman LO Mesenchymal stem cell treatment for chronic renal failure. Stem Cell Res Ther 2014;5:83. 25158205 10.1186/scrt472 PMC4097822

[7] Mak JC Acupuncture in osteoporosis: more evidence is needed. Acupunct Med 2015;33:440–1. 26508661 10.1136/acupmed-2015-010983

[8] Ni Y Shi W Xu X Acupuncture treatment for 34 cases of epiphora with dysfunction of lacrimal duct. J Tradit Chin Med 2002;22:31–2. 11977517

[9] Yang JL Zhang R Du L Clinical observation on the neurotransmitters regulation in patients of insomnia differentiated as yang deficiency pattern treated with warm acupuncture and auricular point sticking therapy. Zhongguo Zhen Jiu 2014;34:1165–8. 25876342

[10] Zhao LH Effects of warm needle moxibustion on bone mass density and biochemical indexes of bone metabolism in patients of postmenopausal osteoporosis. Zhongguo Zhen Jiu 2008;28:897–900. 19127918

[11] Tsui ML Cheing GL The effectiveness of electroacupuncture versus electrical heat acupuncture in the management of chronic low-back pain. J Altern Complem Med 2004;10:803–9. 10.1089/acm.2004.10.803 15650469

[12] Yang L Tan JY Ma H Warm-needle moxibustion for spasticity after stroke: a systematic review of randomized controlled trials. Int J Nurs Stud 2018;82:129–38. 29631145 10.1016/j.ijnurstu.2018.03.013

[13] Ishimura K Shinohara S Kitade T Clinical efficacy of electrical heat acupuncture. Am J Acupunct 1993;21:13–8.

[14] Chen HZ, Editor-in-Chief. Practical Internal Medicine, 12th Edition. Beijing People's Health Publishing House; 2005; 2078–2094.

[15] Greenwood SA Koufaki P Mercer TH Effect of exercise training on estimated GFR, vascular health, and cardio respiratory fitness in patients with CKD: apilotrandomized controlled trial. Am J Kidney Dis 2015;65:425–34. 25236582 10.1053/j.ajkd.2014.07.015

[16] Yang L Tan JY Ma H Warm-needle moxibustion for spasticity after stroke: a systematic review of randomizes controlled trials. Int J Nurs Stud 2018;82:129–38. 29631145 10.1016/j.ijnurstu.2018.03.013

